# A Standardized workflow for *Orthohantavirus* production, detection, and antiviral screening

**DOI:** 10.1186/s12985-026-03136-y

**Published:** 2026-03-21

**Authors:** Hannah S. Schwarzer-Sperber, Tamara Mussfeldt, Julia Boesner, Tina Dluzak, Kathrin Sutter, Roland Schwarzer

**Affiliations:** 1 Institute for the Research on HIV and AIDS-Associated Diseases (HIV-AAD), University Hospital Essen, University Duisburg-Essen, Essen, Germany; 2 Institute of Virology, University Hospital Essen, University Duisburg-Essen, Essen, Germany

## Abstract

**Background:**

*Orthohantaviruses* are zoonotic RNA viruses that cause hemorrhagic fever with renal syndrome and hantavirus cardiopulmonary syndrome. Their slow replication, lack of cytopathic effects, and variable biosafety requirements have long hindered the standardization of infection assays and antiviral testing. While serological and pseudotype-based systems enable high-throughput screening, scalable assays that directly quantify full-cycle *Orthohantavirus* infection remain limited.

**Results:**

Here, we present a modular BSL-2 workflow that integrates standardized virus stock generation with complementary infection-quantification readouts. The platform combines (i) titration-guided pooling of daily supernatants to produce high-titer virus stocks without ultracentrifugation, (ii) a cross-reactive monoclonal antibody recognizing conserved nucleocapsid epitopes across multiple *Orthohantavirus* species, and (iii) three quantitative detection methods: qPCR, in-cell ELISA, and intracellular flow cytometry. Flow cytometry was used as the primary readout because it enables rapid, quantitative, single-cell detection of infection and can be readily integrated with multiparametric analyses. Using this system, we quantified the antiviral effects of rottlerin and observed distinct dose‒response profiles among Puumala, Tula, and Prospect Hill viruses.

**Conclusion:**

Our workflow delivers a practical, reproducible, and scalable toolkit for comparative *Orthohantavirus* studies, enabling quantitative infection analysis and small-molecule testing under standard laboratory conditions.

**Supplementary Information:**

The online version contains supplementary material available at 10.1186/s12985-026-03136-y.

## Introduction

Orthohantaviruses are enveloped, negative-sense RNA viruses of the family Hantaviridae. They are primarily associated with rodents, although shrews, moles, and bats can also serve as hosts. Several *Orthohantavirus* species infect humans and cause severe disease. In Eurasia, they typically lead to hemorrhagic fever with renal syndrome (HFRS), whereas American species can cause hantavirus cardiopulmonary syndrome (HCPS), which has high fatality rates [1]. Their trisegmented genome (L, M, and S segments) encodes the RNA-dependent RNA polymerase (RdRP), glycoproteins (Gn/Gc), nucleocapsid protein (NP) and nonstructural protein (NSs) [1]. The latter however is species specific and is found only in *Orthohantaviruses* carried by rodents in the family Cricetidae [1].

Despite their public health importance and a significant interest in *Orthohantavirus* biology, comprehensive experimental work on *Orthohantaviruses* is hampered by numerous experimental challenges. Compared with many RNA viruses, they replicate relatively slowly, often do not produce overt cytopathic effects in cell culture, and have historically required complex titration assays (e.g., plaque/focus forming, CCID₅₀), which are time-consuming and labor intensive [2, 3].

Therefore, reliable quantification of *Orthohantavirus* infection is a key bottleneck in both mechanistic virology and antiviral screening. While RT-qPCR of viral RNA offers high sensitivity, it measures bulk viral genomes and does not resolve the proportion of infected cells or heterogeneous infection kinetics. Immunoassays (e.g., ELISA, immunofluorescence, flow cytometry, and in-cell western) have been developed for *Orthohantaviruses*, but protocols often lack broad cross-species validation or standardization [4–6].

To address these limitations, we developed a BSL-2-compatible workflow tailored for Old-World *Orthohantaviruses* that integrates three core components: (i) a streamlined virus stock generation protocol based on daily harvest pooling and titration-guided selection; (ii) validation of a broadly cross-reactive monoclonal antibody recognizing conserved NP epitopes across three *Orthohantavirus* species; and (iii) benchmarking of three quantitative readouts—qPCR, in-cell ELISA and intracellular flow cytometry. As a proof of principle, we applied this platform to comparative antiviral testing via the broad-spectrum compound rottlerin, a cell-permeable PKC inhibitor with well-described virus entry-inhibiting effects [7].

This workflow provides a standardized, high-resolution toolkit for *Orthohantavirus* research, offering technical accessibility, quantitative precision and translational relevance for antiviral discovery under routine BSL-2 conditions.

## Materials and methods

### Cell lines and culture conditions

VeroE6 cells (ATCC CRL-1586) and HEK293T cells (ATCC CRL-11268) were maintained in DMEM +/+, which consisted of Dulbecco’s modified Eagle’s medium (DMEM; Gibco) supplemented with 10% fetal bovine serum (FBS; Capricorn Scientific) and 1% penicillin‒streptomycin (Gibco). The cells were incubated at 37 °C in a humidified atmosphere with 5% CO₂. For all infection and transfection assays, cells were seeded at 70% confluence and used at low passage numbers.

### Production of viral stocks

*Orthohantavirus* stocks of Puumala virus (PUUV, strain Sotkamo), Tula virus (TULV, strain Moravia), and Prospect Hill virus (PHV, strain PH-1 ) were produced in VeroE6 cells under biosafety level 2 (BSL-2) conditions. Typically, 8 × 10⁶ cells were seeded into T75 flasks and infected the following day with 100–200 µl of virus stock in 2 ml of DMEM containing 2% FBS and no antibiotics (DMEM +/–). To ensure uniform exposure of the monolayer to the inoculum, the flasks were gently tilted every 15 min for 1 h. Subsequently, 5 ml of DMEM +/– were added to each flask. From day 4 to day 12 postinfection, culture supernatants were collected daily, completely replaced with fresh DMEM +/–, and clarified by low-speed centrifugation (2,000 × g, 10 min, room temperature (RT)) to remove cellular debris. All the supernatants were stored at − 20 °C until further processing. After the final harvest, daily supernatants were titrated by flow cytometry, and the highest-titer fractions (typically days 6–9 postinfection) were pooled and aliquoted to generate working virus stocks. No ultracentrifugation or additional concentration steps were needed. The final virus stocks were stored at − 80 °C and thawed only once prior to use.

### Virus concentration by ultrafiltration

Clarified supernatants were transferred into 50–100 kDa MWCO centrifugal filtration units (Amicon; Sartorius AG) and centrifuged at ~ 4,000 × g and 4 °C until the volume was reduced to 1–3 mL (per unit). When multiple units were processed in parallel, the retentates were combined and gently mixed by pipetting. The concentrate was either used immediately or aliquoted and snap-frozen at − 80 °C (with a single freeze–thaw cycle only).

### Virus concentration via tabletop centrifugation

As an ultracentrifuge-free alternative, the virus was pelleted in a refrigerated tabletop microcentrifuge (fixed-angle rotor). For that purpose, aliquots of clarified supernatant (typically 1.5–2.0 mL per microfuge tube) were spun for 15–45 min at ~ 16,100 × g and 4 °C. The supernatants were carefully removed; the pellets were typically not visible but present. The pellets were immediately resuspended in the desired medium (e.g., DMEM + 2% FBS) at ~ 50–200 µL per tube, pooled, gently mixed, aliquoted, and stored at − 80 °C.

### Virus titrations

For infection assays, 30,000 VeroE6 cells were seeded in 96-well flat-bottom plates and infected with defined volumes of virus-containing supernatant diluted in a total volume of 100 µl of DMEM +/+. Unless otherwise stated, infection was allowed to proceed for 72 h before immunofluorescence staining and flow cytometry analysis.

### Flow cytometry-based detection of infection

Flow cytometry was used as the primary method for quantifying *Orthohantavirus* infection. Infected cells were harvested via TrypLE Express (Thermo Fisher Scientific), washed in PBS, and fixed with 100 µl of 4% paraformaldehyde for 15 min at room temperature. The cells were then permeabilized with 100 µl of 0.1% Triton X-100 in PBS (permeabilization buffer, PB) for 10 min, followed by blocking in PBS containing 2% FBS for 30 min. A commercial fixation and permeabilization reagent (Cyto-Fast Fix/Perm Buffer Set, Biolegend) was used according to the manufacturer instructions for benchmarking.

Staining was performed using primary antibodies against the *Orthohantavirus* nucleocapsid protein N (A1C5-C, Progen, unless otherwise specified) or glycoprotein Gc (manufacturer and catalog numbers are provided in the Results section), followed by Alexa Fluor (AF) 647–conjugated secondary antibodies (Thermo Fisher Scientific). Primary antibodies were typically used at a 1:100 dilution in PB (50 µl) and incubated for 30 min at RT, and secondary antibodies were used at a 1:400 dilution in PB (100 µl) and incubated for 30 min at RT. Finally, the cells were resuspended in 100 µl of FACS buffer (PBS + 2% FBS) and analyzed on a BD LSR II cytometer.

Gating included FSC/SSC-based dead cell exclusion (calibrated in VeroE6 cells by Zombie Aqua dye-exclusion staining; BioLegend) and singlet discrimination using FSC-W/FSC-H. Infected cells were defined as AF647⁺ populations. A minimum of 10,000 live singlet events were acquired per sample.

### Antibody validation and epitope analysis

To evaluate the specificity and cross-reactivity of commercially available anti-N and anti-Gc antibodies, HEK293T cells were transfected with constructs encoding either N-YFP [10] or GnSP-YFP-Gc [11] fusion proteins via polyethylenimine (PEI, Tocris) following the manufacturer’s protocol. At 48 h posttransfection, the cells were processed as described above and stained with the appropriate primary and secondary antibodies. Flow cytometry analysis was performed to assess the AF647 signal intensity relative to YFP fluorescence.

In addition, amino acid sequences of the N protein region recognized by the A1C5-C monoclonal antibody [8] were aligned across PUUV, TULV, and PHV via Clustal Omega (EMBL-EBI) to assess epitope conservation.

### RNA extraction and quantitative PCR (qPCR)

For comparative analysis by qPCR, infected cells were harvested at 72 h post infection via TrypLE Express as described above, then washed with PBS, pelleted, and stored at − 80 °C until RNA extraction. Total RNA was isolated via the RNeasy Mini Kit (Qiagen) according to the manufacturer’s instructions. Reverse transcription was performed with HiScript III RT SuperMix for qPCR + gDNA wiper (Vazyme). The qPCRs were prepared via Luna Universal qPCR Master Mix (New England Biolabs) and run on a qTOWER 2.0 cycler (Analytik Jena). The following primers targeting the viral S segment and β-actin were used: PUUV F “GARRTGGACCCRGATGACGTTAA”, PUUV R “CCKGGACACAYCATCTGCCAT”, b-actin F “GGT GGC TTT TAG GAT GGC AAG”, and b-actin R “ACT GGA ACG GTG AAG GTG ACA G”. Viral RNA levels were normalized to those of β-actin via the ΔCT method.

### In-cell ELISA

In-cell ELISA was performed essentially as previously described for other virus families [13, 14]. Briefly, VeroE6 cells in 96-well plates were fixed with 100 µl of 4% paraformaldehyde for 2 h at RT, followed by permeabilization with 0.1% Triton X-100 (200 µl/well, 30 min at RT). Cells were then incubated with 200 µl/well blocking solution for 1–2 h at RT before addition of the primary anti-N protein antibody (A1C5-C [8], Progen; unless indicated otherwise, 1:300 diluted in 50 µl/well blocking solution). Primary antibody incubation was carried out for 2 h at RT or overnight at 4 °C. After washing, cells were incubated with a goat anti-mouse secondary antibody (Dianova, 115-035-003; 1:2000 in blocking solution, 50 µl/well) for 1–2 h at RT. The secondary antibody was aspirated and wells were washed four times with 250 µl/well wash buffer. 3,3’,5,5’-tetramethylbenzidine (TMB) substrate was then added, and absorbance was measured at 450 nm with 620 nm as reference using a microplate reader (Tecan Infinite 200 Pro). Signals were background-subtracted using uninfected control wells.

### Antiviral compound testing

Rottlerin (MedChemExpress), a polyphenolic compound with previously reported broad-spectrum antiviral activity, was tested in infection inhibition assays. A total of 3 × 10^4^ VeroE6 cells were pretreated with serial dilutions of rottlerin (100 µl) for 1 h prior to infection with PUUV, TULV, or PHV (MOI ~ 0.4, based on virus titration as presented in Fig. 2). After 72 h, infection levels were quantified via flow cytometry as described above. All titrations were normalized by expressing infection levels of each inhibitor-treated sample in percent of the respective mock infection (infected and solvent treated only). IC₅₀ values were calculated by fitting the data to a four-parameter variable slope model in GraphPad Prism (v9): Y = Bottom + (Top − Bottom)/(1 + (IC₅₀/X)^HillSlope). Statistical differences in the IC₅₀ values were assessed via the extra sum-of-squares F test.

### Z’ factor calculation

To quantify assay performance for the rottlerin dose–response, we calculated the screening Z′ factor as a measure of signal window and variability [9].

The Z′ factor was calculated as: ${Z}^{{\prime}}=1-\frac{3({\sigma}_{+}+{\sigma}_{-})}{\left|{\mu}_{+}-{\mu}_{-}\right|}$

​where µ_−_ and σ_−_ denote the mean and standard deviation (SD) of the control sample, and µ_+_ and σ_+_​ denote the mean and SD of the maximal response condition. Z′ values range from < 0 to 1, with higher values indicating better assay separation and robustness.

### Data analysis and statistics

All the experiments were conducted in biological triplicates unless otherwise noted. Flow cytometry data were analyzed via FlowJo (v10.8, BD Biosciences). Graphs were drawn, and statistical analyses were performed via GraphPad Prism (Dotmatics). The data are presented as the means ± standard error of the mean (SEMs) unless otherwise indicated. For statistical comparisons, one-way ANOVA or nonparametric tests were applied as appropriate; p values < 0.05 were considered statistically significant.

## Results

### Comparative evaluation of quantitative infection assays

**Fig. 1:**
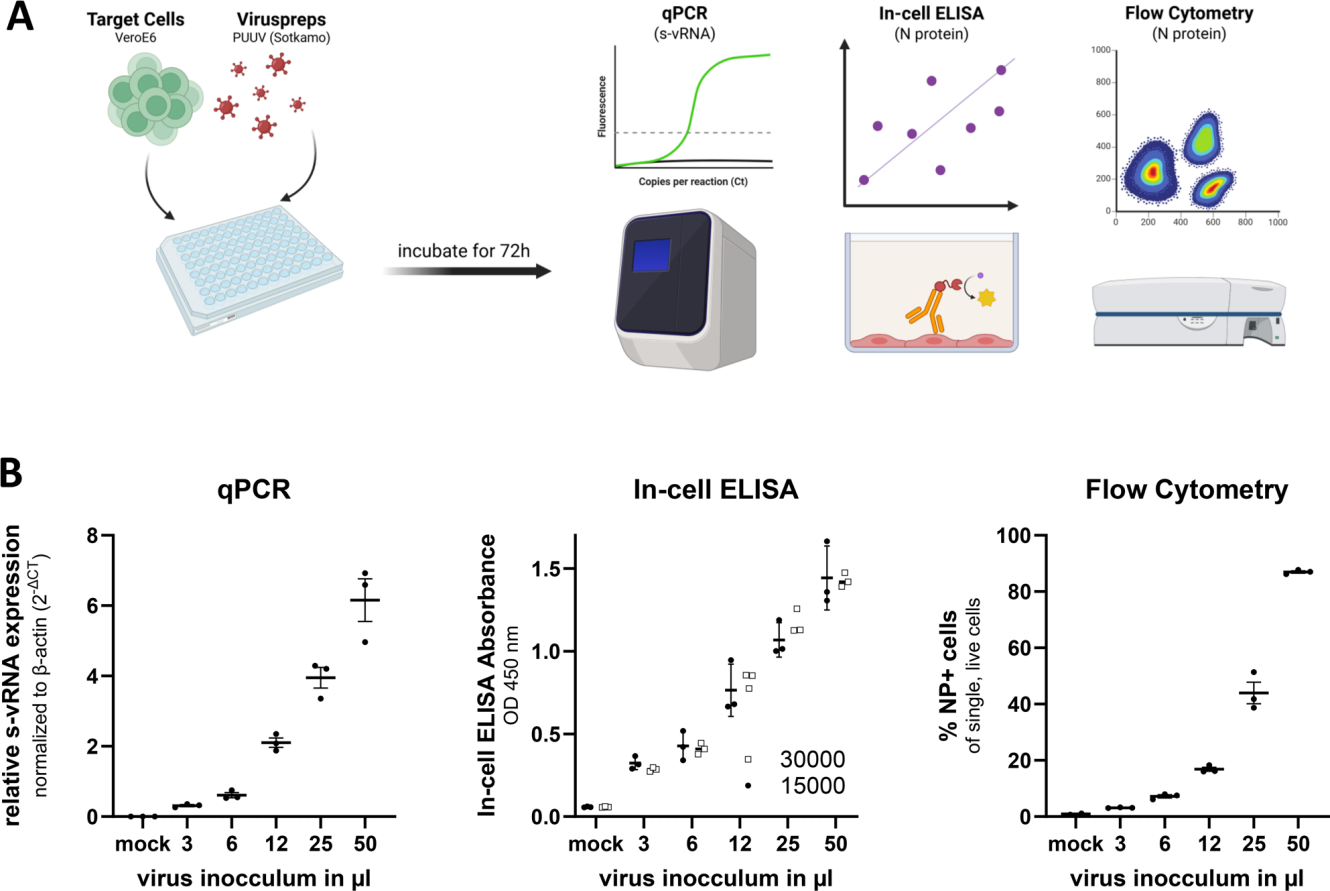
Comparison of three independent assays for the quantification of *Orthohantavirus* infections. (**A**) Experimental workflow. VeroE6 cells were seeded in a 96-well plate and infected with various concentrations of *Orthohantavirus*. After a 72-hour incubation period, infected cells were analyzed via three distinct quantification assays: In-cell ELISA, qPCR and flow cytometry. (**B**) Representative experimental results. qPCR data are presented as viral RNA levels normalized to β-actin expression. The in-cell ELISA data are displayed as raw absorbance values at OD=450 nm. The gray and black points indicate infections of 30000 or 15000 cells, respectively. Flow cytometry data indicate AlexaFluor 647-positive (A647⁺), live single-cell populations. The data represent the means ± standard deviations from three independent experiments (n = 3)

To establish a quantitative framework for assessing *Orthohantavirus* infection in vitro, we evaluated three distinct detection methods: quantitative PCR (qPCR), in-cell ELISA (icE), and intracellular flow cytometry (IFC). These approaches differ in resolution, readout type, and throughput and were systematically compared using *Orthohantavirus*-infected VeroE6 cells to identify suitable methods for downstream assay development (Fig. 1). For all three assays, VeroE6 cells were infected with identical serial dilutions of PUUV, the most common *Orthohantavirus* species in western and central Europe, and incubated for 72 h prior to virus quantification.

#### qPCR enables robust detection of *orthohantavirus* replication across broad ranges of viral inputs

First, we performed qPCR, which remains the benchmark for the quantification of *Orthohantavirus* genomic RNA. To that end, the cells were harvested after infection, and dry cell pellets were frozen. Then, the mRNA was extracted, reverse transcribed to cDNA, and analyzed via SYBR Green-based qPCR targeting the viral S segment, while β-actin served as a housekeeping gene for normalization. A clear, dose-dependent increase in viral RNA was observed across the titration series (Fig. 1B, left panel), confirming the method’s sensitivity and reproducibility.

#### In-cell ELISA provides a protein-level readout and maintains a consistent titration response across different cell seeding densities

Next, we assessed the feasibility of an ELISA-based format that targets the intracellular N protein. Here, VeroE6 cells were seeded at 15,000 or 30,000 cells per well and infected with a dilution series of PUUV. After 72 h, the cells were fixed, permeabilized, stained with an anti-N protein antibody followed by an HRP-conjugated secondary antibody, and developed with TMB substrate. The OD₄₅₀ values increased proportionally with virus input at both cell densities, with the HRP signals being nearly superimposable (Fig. 1B, center panel), indicating robustness to seeding variation. This assay is easy to implement, scalable, and requires no specialized instrumentation, making it a viable high-throughput option.

#### Flow cytometry enables single-cell-resolution quantification of intracellular viral proteins and reveals proportional infection rates across virus inputs

Finally, we adopted a flow cytometry-based infection assay that detects intracellular N protein at the single-cell level. To that end, infected cells were fixed, permeabilized, and stained for infection via a primary N protein antibody and an AF647-conjugated secondary antibody. Flow cytometric analysis was used to quantify infection as the percentage of AF647⁺ cells among live, single-cell events. The resulting data revealed distinct separation between the infected and uninfected populations (Supplementary Fig. S1), whereas the infection frequency increased in a virus input–dependent manner (Fig. 1B, right panel). We then compared assay performance across qPCR, flow cytometry, and in-cell ELISA using the Z’ factor [9] calculated between uninfected controls and maximally infected conditions. qPCR showed acceptable separation (Z’ = 0.49), whereas flow cytometry and in-cell ELISA showed excellent performance with Z’ values of 0.96 and 0.95, respectively.

As the only single-cell-resolved assay among those tested, flow cytometry enables discrimination of infected and uninfected populations and robust quantification across inputs, supporting its selection for further optimization.

### Antibody validation and optimization of flow cytometry-based *Orthohantavirus* protein detection

Flow cytometry is a powerful tool for single-cell detection of *Orthohantavirus* infection, but its reliability depends critically on the specificity and performance of the antibodies used. We therefore validated a panel of commercially available antibodies against the viral nucleocapsid protein (N) and glycoprotein Gc and systematically optimized the staining conditions.

#### Commercial antibodies against the N and Gc proteins specifically detect their targets in YFP-tagged expression systems

**Fig. 2:**
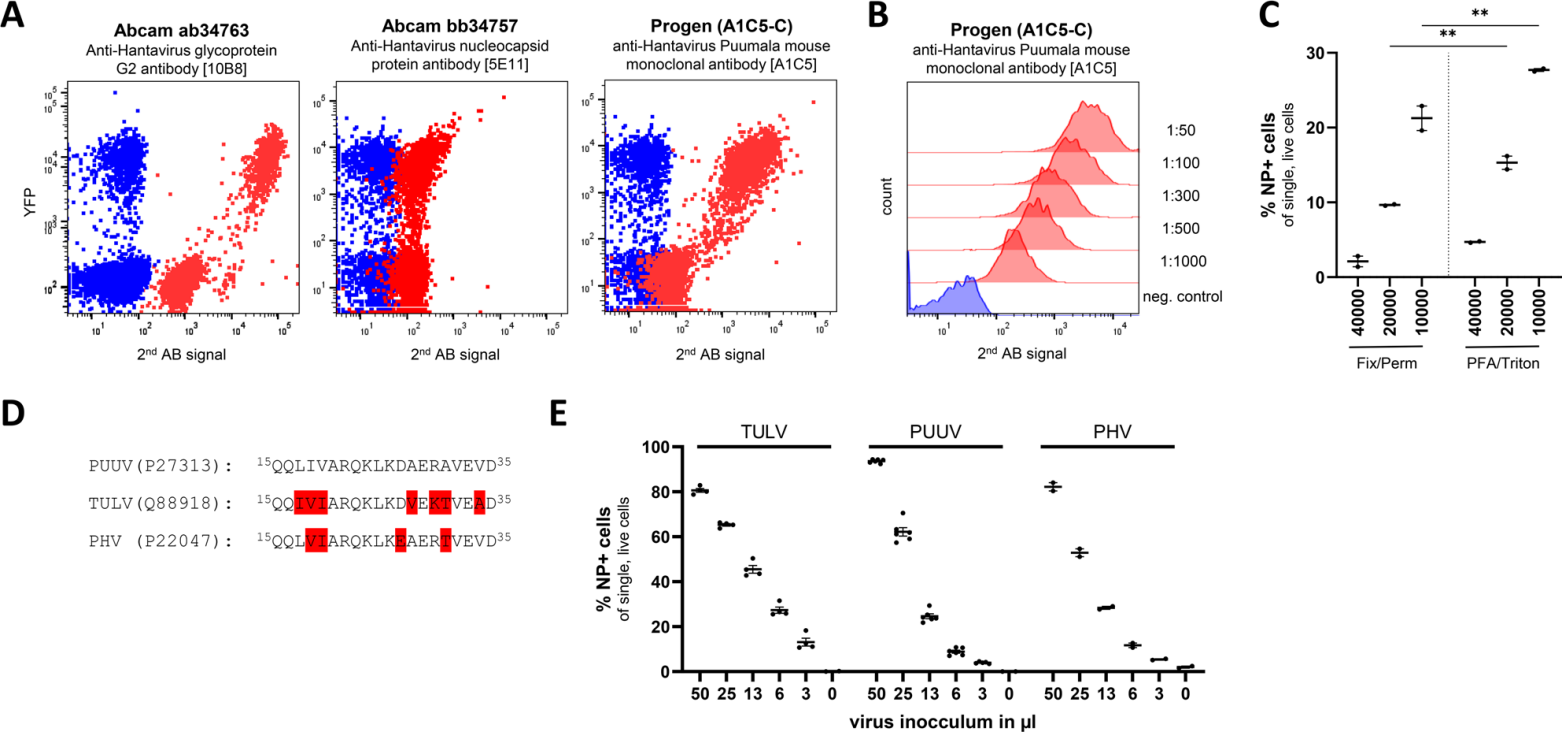
Antibody validation and optimization for flow cytometry-based detection of *Orthohantavirus* infection. (**A**) Evaluation of commercially available antibodies targeting viral structural proteins. HEK293T cells were transfected with either N-YFP [10] or GnSP-YFP-Gc [11] constructs and stained with the indicated antibodies specific to the N or Gc protein, followed by incubation with AlexaFluor 647-conjugated secondary antibodies. Dot plots show AlexaFluor 647 fluorescence (x-axis) versus YFP expression (y-axis). The blue cells represent the transfected controls stained without a secondary antibody. (**B**) N-YFP-transfected HEK293T cells were stained with the anti-N protein antibody A1C5-C and analyzed by flow cytometry. The histograms show the Alexa Fluor 647 signals of samples stained with different primary antibody dilutions. (**C**) Optimization of the staining protocol. Infected VeroE6 cells were subjected to different fixation/permeabilization methods (see Materials and Methods for details) and varying numbers of cell inputs to assess their impact on infection levels and detection sensitivity. Infection was measured as the percentage of A647⁺ cells. Significance was tested via two-way ANOVA, ** P ≤ 0.01. (**D**) Amino acid sequence alignment of the N protein region recognized by the A1C5-C antibody across three *Orthohantaviruses*: PUUV, TULV, and PHV. The alignment highlights sequence variation in the epitope region with PUUV as the consensus sequence. (**E**) Quantification of infection rates across a range of virus inputs for PUUV, TULV and PHV

To evaluate antibody specificity and cross-reactivity, HEK293T cells were transfected with plasmids encoding either N-YFP [10] or GnSP-YFP-Gc [11] fusion proteins. The cells were then stained with anti-N (Abcam ab34757, Progen A1C5-C [8]) or anti-Gc antibodies (Abcam ab34763) followed by AF647-conjugated secondary antibodies. Flow cytometry analysis revealed strong AF647 signals in YFP⁺ cells and minimal signals in nontransfected or secondary-only controls (Fig. 2A) for the Progen N protein and Abcam Gc antibodies but not the Abcam N protein antibody. This confirmed that ab34763 and A1C5 specifically recognized their respective targets under intracellular staining conditions and could be used for infection detection via flow cytometry. On the basis of its excellent performance and cost-effectiveness, we selected the Progen anti-N antibody for all subsequent experiments.

#### Titration of the anti-N antibody identified the optimal working concentrations

To determine the optimal experimental conditions, N-YFP-transfected HEK293T cells were stained with serial dilutions of the anti-N antibody. Flow cytometry histograms revealed a clear decline in the AF647 signal with increasing antibody dilution, whereas background staining remained low across conditions (Fig. 2B, Supplementary Fig. S2). On the basis of these results, a dilution of 1:100 was selected for all subsequent experiments.

#### Fixation and permeabilization, but not seeding density, impact detection sensitivity

To further assess the robustness of the staining protocol, we systematically varied the fixation/permeabilization conditions and the number of cells per sample. To that aim, PUUV-infected VeroE6 cells were processed under various conditions and stained with the optimized anti-N antibody. Both the absolute infection rates and the background levels were influenced by the protocol conditions, with 4% paraformaldehyde fixation and 0.1% Triton X-100 permeabilization yielding significantly better results (Fig. 2C). The assay maintained consistent performance across a range of cell input numbers, supporting its scalability.

#### The epitope recognized by the A1C5-C antibody is conserved across PUUV, TULV, and PHV

To assess the potential for broader applicability, we performed a sequence alignment of the N protein region recognized by the A1C5-C antibody across PUUV, TULV, and PHV. The epitope was highly conserved, with only minor amino acid variation relative to the PUUV consensus (Fig. 2D), suggesting that the antibody could be suitable for cross-strain detection in *Orthohantavirus* studies.

#### Flow cytometry-based detection using the validated anti-N antibody enables robust quantification of infection across multiple virus species

To confirm the cross-reactivity of the antibodies used, VeroE6 cells were infected with PUUV, TULV, or PHV at increasing doses and analyzed by flow cytometry. The percentage of AF647⁺ cells correlated with virus input for each species, and consistent detection was achieved across all three viruses (Fig. 2E). These results demonstrate the versatility and sensitivity of the antibody-based flow cytometry assay and support its use for multispecies comparative analysis.

### Scalable virus production without ultracentrifugation enables robust and reproducible *Orthohantavirus* stock preparation

Standardized virus stocks are essential for reproducible infection assays. Many virus preparation protocols depend on ultracentrifugation, which limits accessibility and scalability. We therefore established a simplified workflow that relies on daily supernatant collection, low-speed clarification, and flow cytometric titration, eliminating the need for specialized equipment.

#### Virus concentrations via bench-top centrifugation or density gradient centrifugation have limited benefit over the use of unprocessed supernatants

**Fig. 3:**
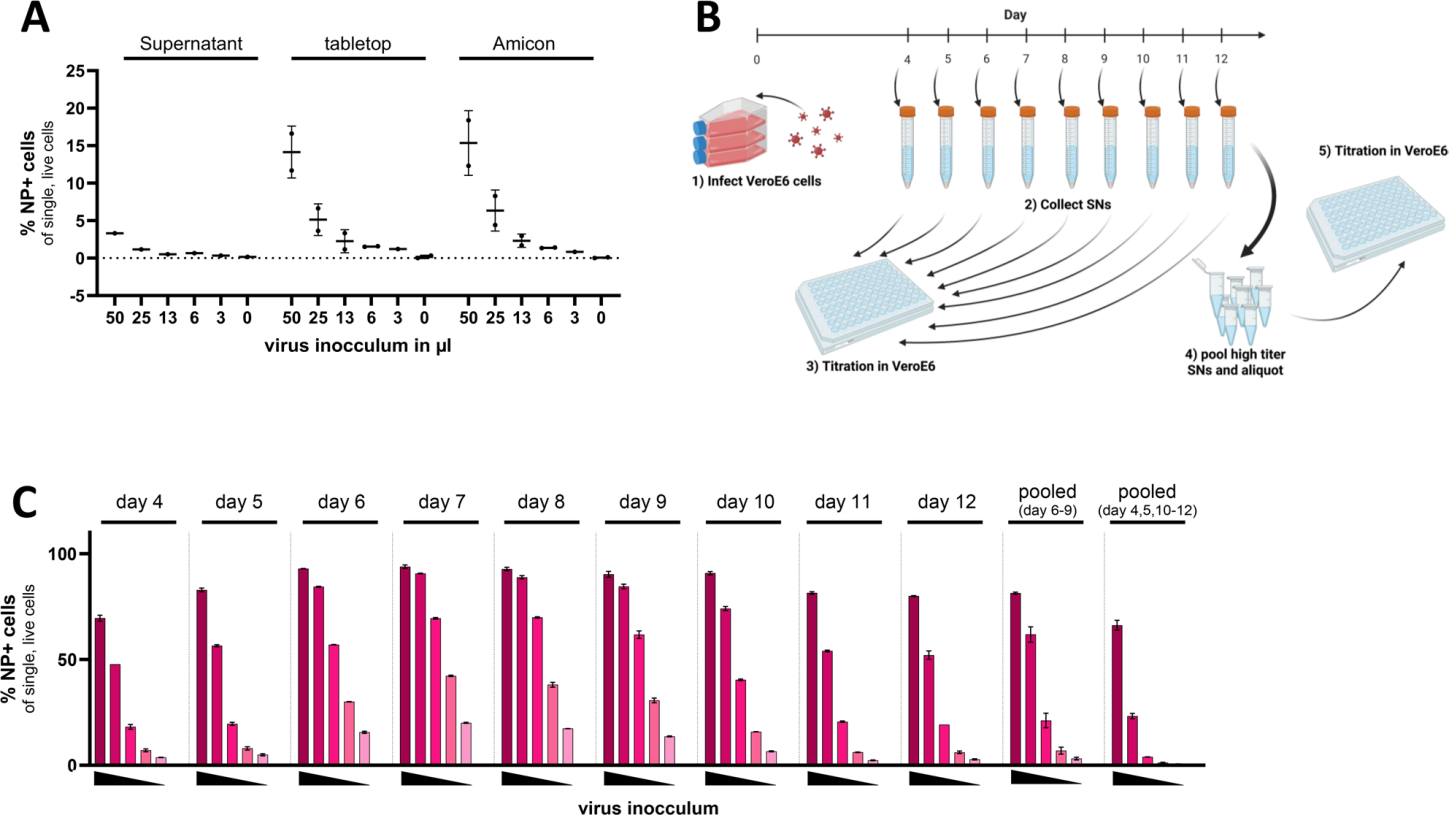
Generation and titration of *Orthohantavirus* stocks. (**A**) Comparison of virus concentration methods. VeroE6 cells were infected with serial dilutions of virus stocks concentrated either by tabletop centrifugation (16,200 × g) or Amicon centrifugal filters. Infection levels were assessed by flow cytometry on the basis of the percentage of live single AF647⁺ cells. (**B**) Workflow for virus stock production without virus enrichment. (1) VeroE6 cells were infected with *Orthohantavirus*. (2) Supernatants (SNs) were collected daily from day 4 to day 12 post infection. (3) The infectivity of each SN was evaluated via titration in VeroE6 cells. (4) High-titer SNs were pooled and aliquoted to generate a working virus stock. (5) The pooled stock was validated by a final titration. (**C**) Representative titration profiles of SNs collected on days 4–12 as well as of pooled virus stocks from days 6-9 or the remaining days. Infection was quantified by flow cytometry on the basis of the proportion of live single AF647⁺ cells. Triangles indicate decreasing input virus volumes: 50, 25, 13, 6, 3, and 0 µl, respectively

To evaluate whether the infectivity of the virus preparation could be improved through different means of ultracentrifugation-independent enrichment, PUUV-containing supernatants were either left untreated, concentrated via Amicon centrifugal filters, or pelleted via bench-top centrifugation at 16,200 × g for 45 min. VeroE6 cells were infected with serial dilutions of each preparation, and infection was quantified via flow cytometry. While concentrated preparations showed a clear increase in infectivity compared with unprocessed supernatants, the fold change was moderate (~ 4.3 bench-top and ~ 4.6 Amicon), especially considering that sample volumes were reduced approximately tenfold during processing (Fig. 3A). Consequently, the total virus yield was lower in the concentrated samples than in the control samples, indicating that enrichment methods result in significant virus loss. These findings support the use of unprocessed, high-titer supernatants as a more efficient and reproducible strategy for generating working virus stocks. If, however, concentration methods are needed to remove, for instance, soluble components such as interferons from virus preparation, tabletop centrifugation achieves similar efficiencies as Amicon-based enrichment.

#### Sequential harvesting reveals batch-specific differences in peak infectivity, requiring titration-guided pooling

To identify the optimal time points for collecting high-titer PUUV stocks, we employed a virus production workflow consisting of infection, daily supernatant harvesting and individual flow cytometric titration of all the supernatants. To that end, we infected VeroE6 cells and harvested the supernatants daily from day 4 to day 12 postinfection (Fig. 3B: steps 1 and 2). Each day’s harvest was then individually titrated via flow cytometry (Fig. 3B: step 3). In this preparation, peak titers were observed between days 6 and 9, with lower infectivity detected in early (days 4–5) and late (days 10–12) samples (Fig. 3C). However, as illustrated in Figure S3, peak infectivity varied considerably across different virus batches, both in timing and magnitude. This variability highlights the importance of empirical titration rather than relying on fixed harvest schedules.

On the basis of these findings, we adopted a routine (schematically summarized in Fig. 3B) in which each daily supernatant is always titrated separately, and only the highest-titer fractions are pooled to generate working virus stocks. Importantly, in the representative experiment at Fig. 3C, pooled supernatants from nonpeak days (4–5 and 10–12) resulted in much lower infection rates, confirming the value of selective pooling for maximizing infectivity.

Of note, this workflow enables reproducible virus production under BSL-2 conditions via only standard laboratory equipment. Moreover, our method avoids the need for ultracentrifugation, requires only basic centrifugation and flow cytometry, and reliably yields functional virus stocks suitable for infection assays and compound screening.

### Application of high-titer virus stocks and flow cytometry assays for antiviral testing

To demonstrate the practical utility of our standardized virus production and flow cytometry-based quantification platform, we evaluated the antiviral efficacy of rottlerin against three *Orthohantavirus* species: PUUV, TULV, and PHV. Rottlerin is a polyphenolic compound previously shown to inhibit the macropinocytosis-dependent entry of PUUV [7], but its broader antiviral activity across *Orthohantaviruses* has not been systematically assessed.

### Flow cytometry enables precise, dose-dependent quantification of rottlerin-mediated inhibition across *Orthohantavirus* species

**Fig. 4:**
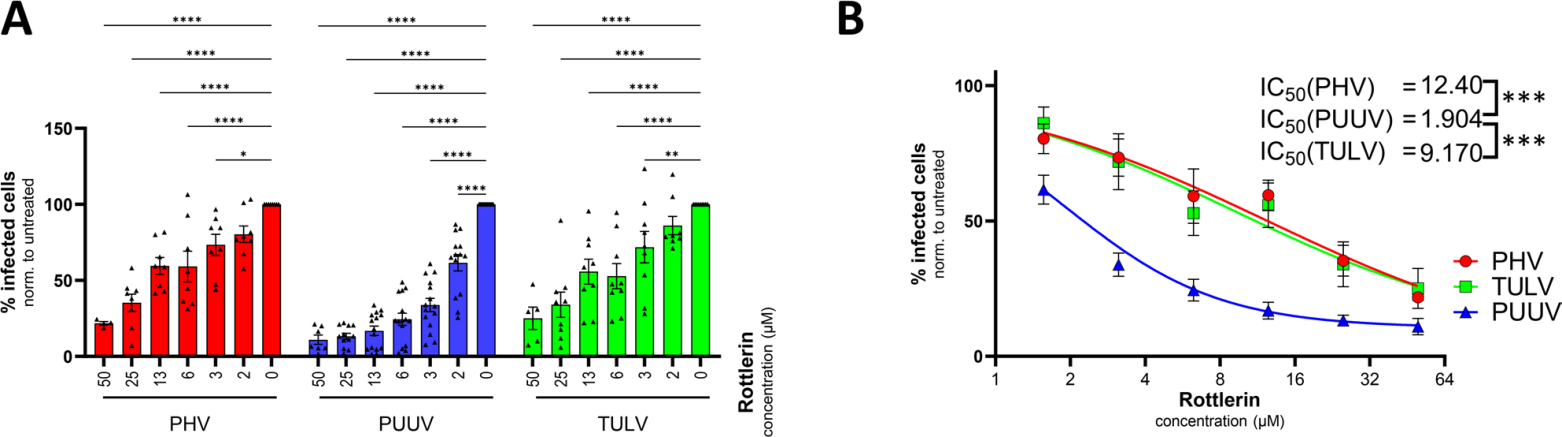
Application of the flow cytometry-based infection assay to antiviral compound testing. (**A**) To assess the antiviral activity of rottlerin, VeroE6 cells were infected with different *Orthohantaviruses* (MOI ~ 0.4) in the presence of increasing concentrations of rottlerin, a natural polyphenolic compound with reported antiviral effects [7]. After 72 h, infection levels were quantified via flow cytometry on the basis of the percentage of NP⁺ live single cells. The bars represent the means ± SEMs from at least three independent experiments. Dots indicate results from individual samples. (**B**) Inhibitor‒response curves were fitted to the data in (A) via the “[Inhibitor] vs. response–Variable slope (four parameters)” function in GraphPad Prism. The model is defined by the equation: Y = Bottom + (Top – Bottom)/(1 + (IC₅₀/X)^HillSlope). IC₅₀ values were statistically compared via the extra sum-of-squares F test. Significant p values are indicated vertically on the plot. Significance is displayed as *p* < 0.05 (*), *p* < 0.01 (**), *p* < 0.001 (***), and *p* < 0.0001 (****)

VeroE6 cells were infected with PUUV, TULV, or PHV in the presence of increasing concentrations of rottlerin. After 72 h, infection rates were quantified via flow cytometry following intracellular staining of the viral N protein. All three viruses showed a clear, concentration-dependent reduction in the percentage of NP⁺ cells, demonstrating that the assay can capture graded antiviral effects across different *Orthohantavirus* species (Fig. 4A). Moreover, these data confirm the antiviral activity of rottlerin against PUUV and extend its observed effect to TULV and PHV. To independently verify that the inhibitory effect is not confined to the flow-cytometric NP readout, we evaluated PUUV infection at a single medium/high rottlerin concentration previously reported to fully suppress macropinocytosis [7], using all three assay formats. Each readout, flow cytometry (NP⁺ cells), in-cell ELISA (N protein), and qPCR (viral RNA), showed a marked and significant reduction compared with the DMSO control (Fig. S4), corroborating the dose–response results presented in Fig. 4.

Finally, we also assessed the performance of our flow cytometry assay using the Z’ factor [9] calculated between the DMSO control and the maximal rottlerin condition, yielding Z’ values of 0.91 (PHV), 0.73 (PUUV), and 0.34 (TULV). This indicates excellent separation for PHV and PUUV, with a smaller but still acceptable signal window for TULV [9].

### Fitted dose‒response curves reveal distinct IC₅₀ values for PUUV, TULV, and PHV

To quantify the inhibitory effect of rottlerin, infection data were modeled via a four-parameter logistic function. Fitted curves revealed differential sensitivity to rottlerin across the three viruses, with PUUV showing the strongest inhibition (lowest IC₅₀). TULV and PHV were significantly less responsive to the rottlerin treatment, resulting in a greater IC₅₀ and a shallower inhibition curve (Fig. 4B). Statistical analysis via the extra sum-of-squares F test confirmed significant differences in the IC₅₀ values (*p* < 0.05), indicating species-specific variation in susceptibility to the compound.

By applying defined virus stocks and standardized infection readouts, we confirmed previous mechanistic insights regarding PUUV entry inhibition and extended the approach to quantify interspecies differences in drug response. This underscores the flexibility of the platform for both hypothesis-driven experiments and broader antiviral screening campaigns.

## Discussion

*Orthohantavirus* research has lacked streamlined, quantitative tools that can be deployed entirely under routine BSL-2 conditions. Here, we present an integrated pipeline to produce highly infectious virus stocks without ultracentrifugation, assess viral stocks via flow cytometry, and quantify infections at single-cell resolution—enabling comparative virologic and antiviral testing across PUUV, TULV, and PHV.

### Flow cytometry is a sensitive, single-cell readout method

Most previous flow cytometry assays targeted new-world *Orthohantavirus*es (e.g., Andes virus and Sin Nombre virus) for titration or entry-inhibitor screens [5, 6]. A very recent study by Menke & Sieben [4] extended the approach to an Old-World virus, PUUV, via automated imaging and flow cytometry. Building on that precedent, we present the first fully standardized, multispecies protocol for three Old-World *Orthohantaviruses*—PUUV, TULV, and PHV. Systematic optimization of fixation/permeabilization, antibody dilution, and cell input, together with a low-cost anti-N monoclonal antibody recognizing a conserved epitope across these viruses (Fig. 2), yielded a broad dynamic range and minimal background. Notably, this antibody also detects the Sin Nombre N protein [12], indicating that the same workflow should translate to New-World hantaviruses, facilitating direct Old- versus New-World comparisons within a single platform.

### Accessible virus production by titration-guided pooling

Standard *Orthohantavirus* preparation procedures often rely on ultracentrifugation. Our workflow instead harvests supernatants daily, titrates each batch by flow cytometry and pools only peak-titer fractions (Fig. 3B-C). Supplementary data (Fig. S3) show that peak infectivity varies between preparations, underscoring the need for empirical titration. Although Amicon filtration or benchtop pelleting modestly increased the titer, overall virus recovery decreased because the volume was reduced approximately tenfold (Fig. 3A). Daily titration plus selective pooling offers the best balance of yield, infectivity and practicality.

### In-cell ELISA as a rapid, high-throughput alternative

Our colorimetric in-cell ELISA adapts the HRP/TMB icELISA concepts established for SARS-CoV-2 [13] and rabies lyssavirus [14] to *Orthohantaviruses*. The assay, which is conducted entirely in 96-well plates and read on a conventional plate reader, is inherently high-throughput, making it ideal for rapid titrations or primary compound screens. The signals scale linearly with virus input and remain stable across moderate changes in cell density (Fig. 1B). Flow cytometry, on the other hand, remains the method of choice when single-cell resolution, absolute virus quantification (e.g., infectious units per ml) or multiplexing is needed. Practically, ELISA provides fast first-pass readout, whereas flow cytometry supplies high-resolution confirmation. A complementary plate-based approach using infrared in-cell Western (ICW) detection of PUUV N protein has been reported by Nusshag et al. [15]. Compared with the infrared imaging-based ICW, our in-cell ELISA however, is optimized for standard plate readers [15]. In addition to ELISA-, IFA-, and flow-based readouts, an enzyme-linked focus-formation assay (FFA) was previously optimized for Hantaan virus (HTNV) and shown to halve turnaround time versus conventional plaque/CCID₅₀ workflows [3]. While the HTNV FFA is undoubtedly faster than classic plaque/CCID₅₅₀ assays and can support antiviral evaluation (e.g., favipiravir, baloxavir acid), it remains an endpoint, overlay-dependent format [3]. Our intracellular flow and in-cell ELISA methods provide earlier, scalable quantification and single-cell or well-level metrics that extend across PUUV, TULV, and PHV.

### Antiviral proof-of-concept: rottlerin

Using standardized virus stocks and flow cytometry, we quantified the activity of rottlerin, which was previously shown to block macropinocytosis-dependent PUUV entry [7]. Rottlerin inhibited PUUV, TULV and PHV in a dose-dependent manner (Fig. 4A), but the IC₅₀ values were species specific: PUUV < TULV ~ PHV (Fig. 4B). These differences highlight both the importance of cross-species testing and the platform’s capacity to resolve nuanced antiviral effects.

### Limitations and outlook

We would like to point out that VeroE6 cells do not fully mimic human target tissues. Importantly, the extent to which *Orthohantaviruses* rely on macropinocytosis and related endocytic routes is virus- and cell-type-dependent. For example, Torriani et al. reported macropinocytosis-associated entry features for both an Old World (HTNV) and a New World hantavirus (ANDV) in airway epithelial cells, while also identifying virus-specific differences in the required regulatory factors of macropinocytosis [16]. In this context, there is currently no simple Old World vs. New World dichotomy for macropinocytosis dependence; rather, pathway usage appears shaped by the viral glycoproteins/receptor interactions and the cellular background [17, 18]. Accordingly, rottlerin sensitivity is consistent with involvement of macropinocytosis-related processes but should not be interpreted as definitive proof of a single-entry route. 

Extending the workflow to primary endothelial or renal cultures (or organoid systems) will enhance physiological relevance. The addition of multiplexed host-marker panels or reporter viruses could provide deeper mechanistic insight, and coupling to automated liquid handling will enable large-scale screens.

### Conclusion

By combining titration-guided virus production, antibody-validated flow cytometry and rapid in-cell ELISA, we provide a practical toolkit that fills a critical methodological gap in hantavirus research. Its adaptability, cross-species compatibility and reliance on standard BSL-2 infrastructure should accelerate both fundamental discovery and translational efforts in hantavirology.

## Supplementary Information

Below is the link to the electronic supplementary material.

Supplementary Material 1.

## Data Availability

All data supporting the findings of this study are available within the paper and its Supplementary Information.
