# Supplementary material for: A Standardized workflow for Orthohantavirus production, detection, and antiviral screening
Source: Virol J. 2026 Mar 21;23:94. doi: 10.1186/s12985-026-03136-y (PMC13064277; doi:10.1186/s12985-026-03136-y)
Supplement: Supplementary file 1 — Supplementary Material 1. [file 12985_2026_3136_MOESM1_ESM.pdf]

SUPPLEMENTARY MATERIAL

A Standardized BSL-2 Workflow for Orthohantavirus Production, Detection, and Antiviral Screening

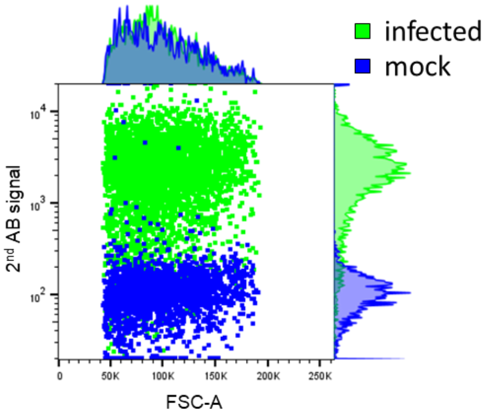

Figure S1: Specific detection of PUUV N protein in infected VeroE6 cells. VeroE6 cells were infected with PUUV for 72 hours and stained with an anti-N antibody followed by AlexaFluor 647-conjugated secondary antibody. Infected cells (green) show elevated A647 signal compared to uninfected controls (blue), confirming specific intracellular detection of the N protein.

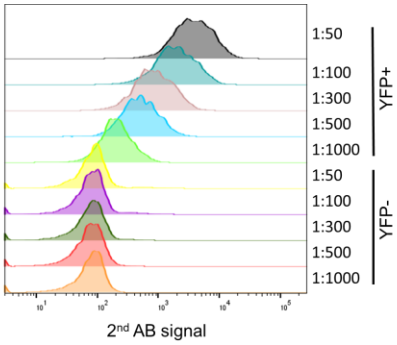

Figure S2: Antibody validation and optimization. HEK 293T cells were transfected with N-YFP (Welke et al., 2022) and stained with A1C5-C, followed by AlexaFluor 647-conjugated secondary antibodies. Histograms show AlexaFluor 647 fluorescence of transfected cells, gated for YFP+ (top) and YFP- (bottom) cells, stained with different dilutions of A1C5-C.

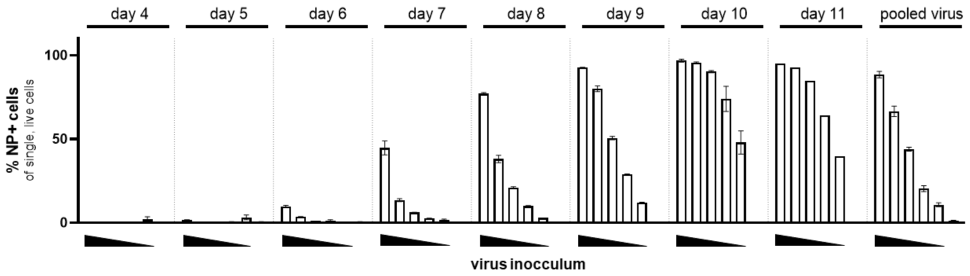

Figure S3: Generation and titration of Orthohantavirus stocks. Representative titration profiles of SNs collected on days 4–12 as well as of pooled virus stocks from day 9-11. Infection was quantified by flow cytometry, based on the proportion of A647⁺, live single cells. Triangles indicate decreasing virus volumes: 50, 25, 13, 6, 3 and 0 µl, respectively.

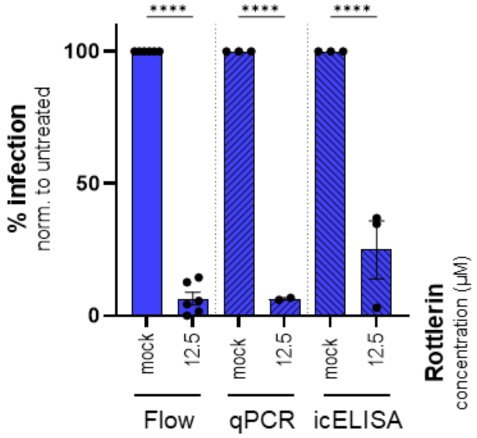

Figure S4: Single-dose cross-assay confirmation of rottlerin-mediated inhibition. VeroE6 cells were infected with PUUV (MOI ~0.4) for 72 h in the presence of 12.5µM Rottlerin. Infection was then quantified in parallel by RT-qPCR (viral RNA), in-cell ELISA (N protein), and flow cytometry (intracellular N staining; %NP⁺ cells). For each assay, values were normalized to the corresponding DMSO control (set to 100%). Bars represent mean ± SEM from 1 experiment. Dots indicate individual samples. All assays were conducted in at least two biological replicates.
